# Optimizing the Use of Serum Immunofixation for the Detection of Monoclonal Components in Hypogammaglobulinemia: Insights From a Tertiary-Care Biochemistry Laboratory

**DOI:** 10.7759/cureus.102571

**Published:** 2026-01-29

**Authors:** Kholoud Krimi, Dounia El Moujtahide, El-Houcine Sebbar, Mohammed Choukri

**Affiliations:** 1 Faculty of Medicine and Pharmacy, Mohammed First University, Oujda, MAR; 2 Central Laboratory, Mohammed VI University Hospital, Bouskoura, MAR

**Keywords:** hypogammaglobulinemia, light-chain abnormalities, monoclonal gammopathy, serum immunofixation electrophoresis, serum protein electrophoresis

## Abstract

Hypogammaglobulinemia is a heterogeneous immunologic abnormality that may result from primary antibody deficiencies, secondary causes, or hematologic malignancies. Although serum protein electrophoresis (SPEP) is routinely used for initial evaluation, reduced γ-globulin levels may mask small monoclonal components, limiting the sensitivity of SPEP alone. Serum immunofixation electrophoresis (IFE), with its superior analytical resolution, may therefore be useful in the diagnostic assessment of hypogammaglobulinemia. The primary objective is to determine the diagnostic yield of systematic serum IFE in adult patients with hypogammaglobulinemia detected by SPEP, defined here as the proportion of patients in whom IFE identified a monoclonal or oligoclonal immunoglobulin abnormality not apparent on SPEP alone. The secondary objectives are to describe the immunochemical patterns identified (monoclonal vs. oligoclonal) and to characterise the detected isotypes (heavy-chain class and light-chain type), including biclonal and light-chain-only profiles. A retrospective descriptive study was conducted over a 12-month period (March 2023-March 2024) at the Central Biochemistry Laboratory of Mohammed VI University Hospital, Oujda, Morocco. Adult patients (≥16 years) presenting with hypogammaglobulinemia, defined as γ-globulin <8 g/L on SPEP, were included. Serum IFE was systematically performed to confirm or exclude the presence of monoclonal immunoglobulin components. Demographic and laboratory data were extracted from the laboratory information system and analyzed descriptively. A total of 261 patients were included (52.9% male; mean age 55.3 years, range 16-82). Most were referred from hematology (61.3%), internal medicine (23.0%), and oncology (15.7%). Serum IFE revealed monoclonal or oligoclonal abnormalities in 63 patients (24.1%), while 198 (75.9%) showed no detectable monoclonal component. Among the 63 abnormal profiles, 59 (93.6%) were monoclonal gammopathies, predominantly of IgG (67.8%) and IgA (30.5%) isotypes. Biclonal gammopathies accounted for 3.1% of cases, and another 3.1% exhibited isolated λ light-chain bands without detectable heavy chains, warranting further urinary immunofixation testing. These findings indicate that nearly one in four patients with hypogammaglobulinemia had a monoclonal component not identifiable by SPEP alone. Systematic serum IFE can therefore improve laboratory assessment by uncovering masked monoclonal components that SPEP may fail to detect. The predominance of IgG and IgA clones, together with the detection of biclonal and light-chain-only patterns, highlights the value of IFE in raising suspicion for clinically relevant plasma-cell and B-cell disorders. Accordingly, these results support considering routine IFE in selected hypogammaglobulinemic patients, particularly when clinical or laboratory features suggest an underlying monoclonal process.

## Introduction

Hypogammaglobulinemia encompasses a heterogeneous group of disorders characterized by a decreased concentration of circulating immunoglobulins (Igs). This reduction may result from primary immunodeficiency syndromes; secondary causes such as lymphoid malignancies, protein loss, or immunosuppressive therapy; or increased catabolism of Igs. From a diagnostic standpoint, the initial biological investigation commonly involves serum protein electrophoresis (SPEP), which identifies a reduced γ-globulin fraction and thus provides a preliminary indication of humoral immune impairment. However, SPEP lacks the resolution to define the immunologic nature of the defect or to detect a monoclonal component potentially masked by the overall decrease in γ-globulins, underscoring the need for more refined diagnostic approaches [1,2].

Igs are antigen-specific glycoproteins essential to the humoral immune response, migrating within the γ-globulin fraction on electrophoresis. Produced predominantly by plasma cells, the terminal differentiation of antigen-stimulated B lymphocytes within the bone marrow, Igs mediate the recognition and neutralization of extracellular pathogens. The plasma half-life of Igs varies by isotype, from several hours (IgE) to several weeks (IgG and subclasses) [3,4]. In adults, hypogammaglobulinemia is defined as a serum γ-globulin concentration below 5 g/L, and its detection should prompt an etiological assessment to determine whether the defect is transient, secondary, or due to a primary antibody deficiency [5]. Identifying the underlying cause is essential to guide appropriate management, including Ig replacement, infection prophylaxis, and surveillance for complications.

Adult-onset hypogammaglobulinemia may be secondary to excessive protein loss (as in nephrotic syndrome or enteropathy), lymphoid malignancies such as chronic lymphocytic leukemia or certain non-Hodgkin lymphomas, or the use of immunosuppressive or antiepileptic medications [6,7]. By contrast, primary antibody deficiencies are intrinsic defects in immune function, the most frequent being common variable immunodeficiency (CVID), which represents the most prevalent symptomatic primary immunodeficiency in adults [8]. CVID is typically diagnosed between the second and fourth decades of life, with a slight female predominance reported in several large cohorts [9]. Its clinical spectrum is remarkably heterogeneous, encompassing recurrent bacterial infections, autoimmune manifestations, lymphoproliferative disorders, and gastrointestinal disease [10,11]. Notably, some patients remain asymptomatic, and hypogammaglobulinemia is detected incidentally during routine electrophoresis or family screening for immune disorders. The immunophenotypic profile in these cases may show diverse abnormalities across B- and T-cell compartments, reflecting the multifactorial pathophysiology of antibody deficiency [12].

In such a complex clinical landscape, serum immunofixation electrophoresis (IFE) plays a pivotal role in detecting and characterizing monoclonal Igs (IgG, IgA, IgM, and κ or λ light chains). IFE provides a higher analytical sensitivity and specificity than standard electrophoresis and is particularly valuable for identifying coexisting monoclonal gammopathies that may remain undetected in hypogammaglobulinemic profiles [12,13]. This is clinically significant, as several hematologic disorders, including multiple myeloma, Waldenström macroglobulinemia, and monoclonal gammopathy of undetermined significance (MGUS), can present with deceptively low γ-globulin levels, potentially leading to diagnostic delay if IFE is omitted [14,15]. Therefore, systematic use of IFE in the evaluation of hypogammaglobulinemia may enhance diagnostic accuracy and allow timely identification of underlying malignant or pre-malignant conditions.

Despite the recognized diagnostic utility of IFE, its systematic prescription in cases of hypogammaglobulinemia remains inconsistent across laboratories and clinical settings. To address this gap, the present study aims to evaluate the clinical relevance and diagnostic yield of serum immunofixation testing in patients with hypogammaglobulinemia identified by SPEP, based on the experience of the Biochemistry Laboratory of Mohammed VI University Hospital, Oujda, Morocco. By assessing the frequency and typology of monoclonal components uncovered by IFE, this work seeks to optimize diagnostic strategies and establish evidence-based recommendations for the rational use of immunofixation in the diagnostic workup of hypogammaglobulinemia.

## Materials and methods

Study design and setting

This retrospective descriptive study was conducted in the Central Biochemistry Laboratory of Mohammed VI University Hospital, Oujda, Morocco, a regional reference center for SPEP and immunologic profiling. The study covered a 12-month period from March 2023 to March 2024. It was based on a review of archived laboratory records, including SPEP and serum IFE files, routinely performed as part of the diagnostic work-up for patients from various hospital departments.

Study population and exclusion and inclusion criteria

Inclusion Criteria

All adult patients (aged ≥ 16 years) who demonstrated hypogammaglobulinemia on SPEP were included in the analysis. Hypogammaglobulinemia was defined as a γ-globulin fraction strictly below 8 g/L. For each eligible patient, serum IFE was systematically performed to confirm or exclude the presence of a monoclonal Ig component as part of the etiological investigation.

Exclusion Criteria

Pediatric patients were excluded, owing to the physiological variability of Ig concentrations during immune system development and incomplete or non-interpretable electrophoresis or immunofixation records.

Laboratory methods

All analyses were performed according to the standard operating procedures in force at the biochemistry laboratory.

SPEP

Protein separation was carried out on agarose gel using a Sebia® electrophoresis system, in strict accordance with the manufacturer’s recommendations. The electrophoretic pattern allowed quantification of the principal serum protein fractions and detection of decreased γ-globulin levels.

Serum IFE

The detection and typing of monoclonal Igs were performed using the Sebia® Hydragel IF K20 kit (Sebia, France). The procedure involves two main steps:

Electrophoretic separation: Serum samples were diluted 1:5 for the IgG track and 1:2 for other tracks using a pH 7.5 buffer containing bromophenol blue. The diluted samples were applied to an agarose gel and subjected to electrophoresis to separate the serum proteins.

Immunoprecipitation and staining: Each gel was incubated with five monospecific antisera (anti-IgG, anti-IgA, anti-IgM, anti-κ, anti-λ; Sebia®), applied at 25 µL per track. After antigen-antibody reaction and washing, precipitated complexes were visualized using acid violet stain. A sixth track (ELP) served as a reference for the overall protein profile, fixed with a proprietary reagent supplied in the kit.

The IFE technique thus enabled the identification and isotyping of monoclonal Igs, distinguishing between IgG, IgA, and IgM heavy chains and κ or λ light chains.

Quality Assurance

All assays were subjected to daily internal quality control and periodic external quality assessment programs to ensure the accuracy, reproducibility, and analytical performance of the electrophoretic and immunofixation procedures. The laboratory participates in national and international quality schemes accredited for clinical chemistry testing.

Data Management and Statistical Considerations

Patient demographic and laboratory data were extracted from the electronic laboratory information system. Descriptive statistics were computed for quantitative and qualitative variables, including age, sex, and hospital department of origin. The prevalence of monoclonal components detected by IFE among hypogammaglobulinemic patients was determined as a proportion of the total cohort.

## Results

Study population and serum immunofixation findings

**Figure 1:**
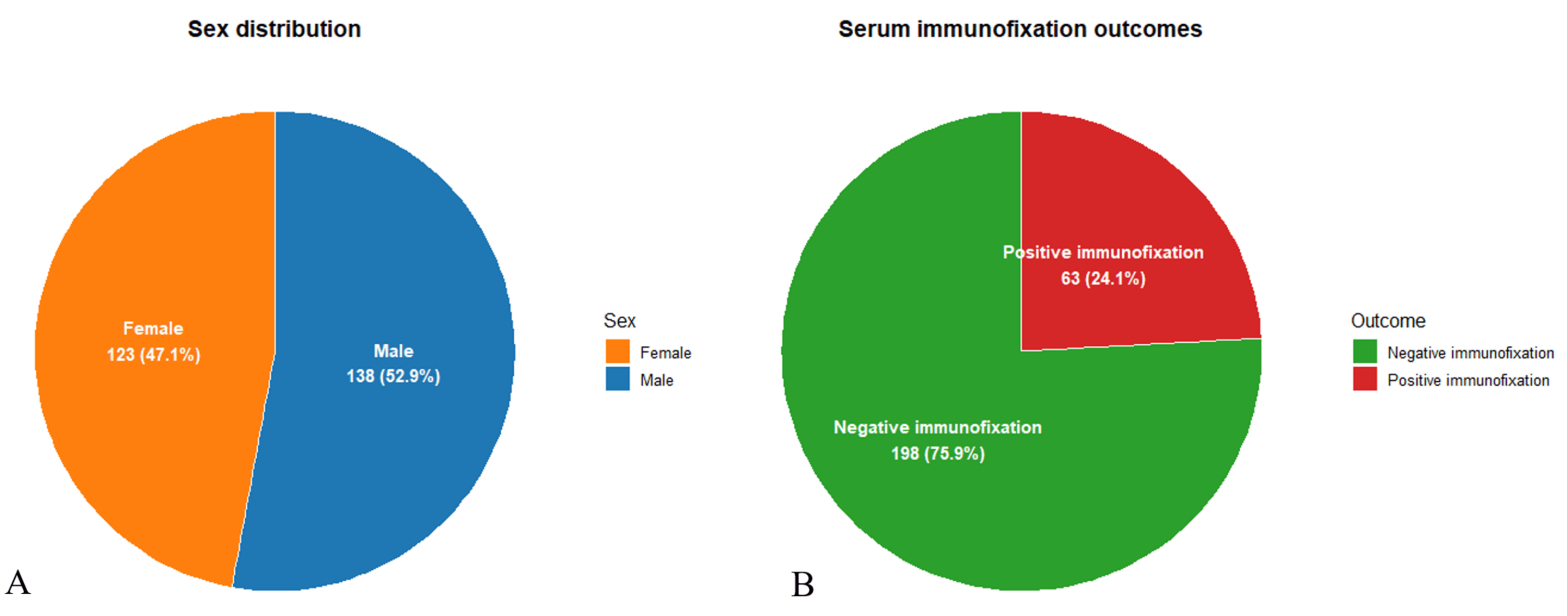
Sex distribution (A) and serum immunofixation outcomes (B). Left: sex distribution (male vs. female). Right: percentage of positive and negative immunofixation results. Figure created by the authors with RStudio (Posit PBC, USA)

After applying the eligibility criteria, 261 adult patients were included in the analysis. The cohort comprised 138 men (52.9%) and 123 women (47.1%) (Figure 1A), with a mean age of 55.3 years (range, 16-82 years). Most patients were referred from the hematology (160 cases, 61.3%), internal medicine (60 cases, 23.0%), and oncology departments (41 cases, 15.7%), highlighting the broad clinical spectrum in which hypogammaglobulinemia is encountered (Table 1).

**Table 1:** Demographic and clinical characteristics of the study population (n = 261).

Characteristic	Number (n)	Percentage (%)	Details / range
Total patients	261	100%	—
Sex			
- Male	138	52.9%	
- Female	123	47.1%	
Age (years)	—	—	Mean = 55.3, range = 16-82 years
Clinical departments of origin			
- Hematology	160	61.3%	
- Internal Medicine	60	23.0%	
- Oncology	41	15.7%	
Definition of hypogammaglobulinemia	—	—	γ-globulin < 8 g/L on SPEP
Follow-up test performed	—	—	Serum immunofixation electrophoresis (IFE)

Figure 1 illustrates the sex distribution and the overall immunofixation outcomes within the study population. A total of 261 adult patients presenting with hypogammaglobulinemia (defined as γ-globulin < 8 g/L on SPEP) and who underwent serum IFE were included in the study. The cohort consisted of 138 men (52.9%) and 123 women (47.1%), with a mean age of 55.3 years (range, 16-82 years). Most cases were referred from the hematology (39.1%), internal medicine (33.3%), and oncology (27.6%) departments, reflecting the heterogeneous clinical settings in which hypogammaglobulinemia is encountered. Out of 261 patients, 63 (24.1%) showed abnormal serum immunofixation profiles, while 198 (75.9%) had negative immunofixation results with no detectable monoclonal or free light-chain (FLC) component (Figure 1B). Among the 63 positive immunofixations, 59 cases (93.6%) corresponded to monoclonal gammopathies, two cases (3.1%) were biclonal gammopathies, and two cases (3.1%) exhibited isolated lambda light-chain bands without a corresponding heavy chain.

These results underscore that nearly one in four patients with hypogammaglobulinemia harbored an underlying monoclonal component detectable only through IFE, which would have been missed by electrophoresis alone.

Distribution of monoclonal isotypes

**Figure 2:**
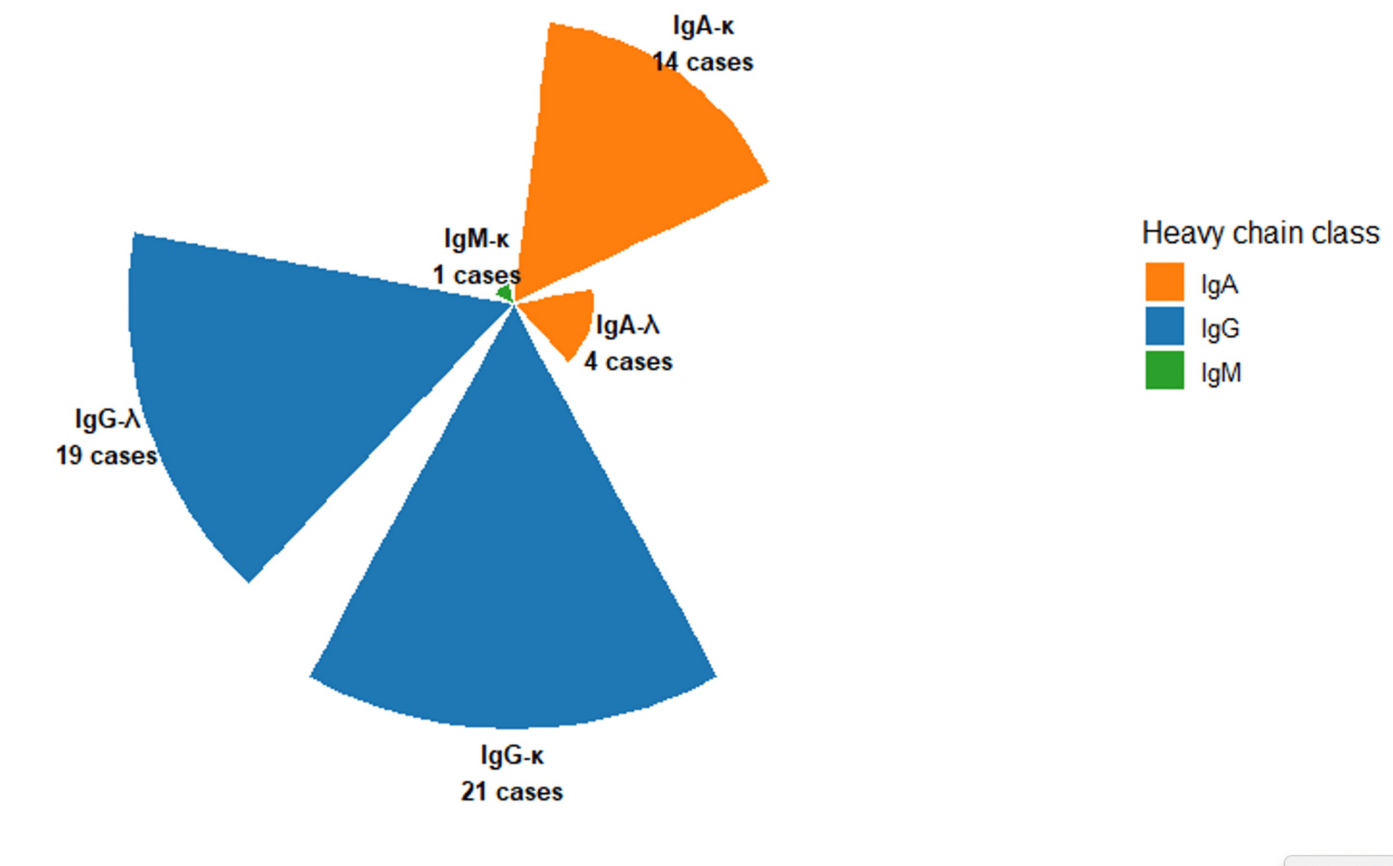
Radial distribution of monoclonal immunoglobulin isotypes (serum immunofixation). Distribution of monoclonal patterns (IgG, IgA, and IgM) detected among patients with hypogammaglobulinemia. Figure created by the authors with RStudio (Posit PBC, USA)

Figure 2 presents the radial distribution of detected monoclonal immunoglobulin isotypes, visually emphasizing the predominance of IgG subclasses. Among the 59 monoclonal gammopathies, IgG was the most frequent heavy chain detected, accounting for 40 cases (67.8%), including 21 IgG-κ (35.6%) and 19 IgG-λ (32.2%) profiles. IgA monoclonalities were observed in 18 cases (30.5%), subdivided into 14 IgA-κ (23.7%) and 4 IgA-λ (6.8%). Only one case (1.7%) involved IgM-κ, and no IgM-λ monoclonality was identified (Figure 2).

**Figure 3:**
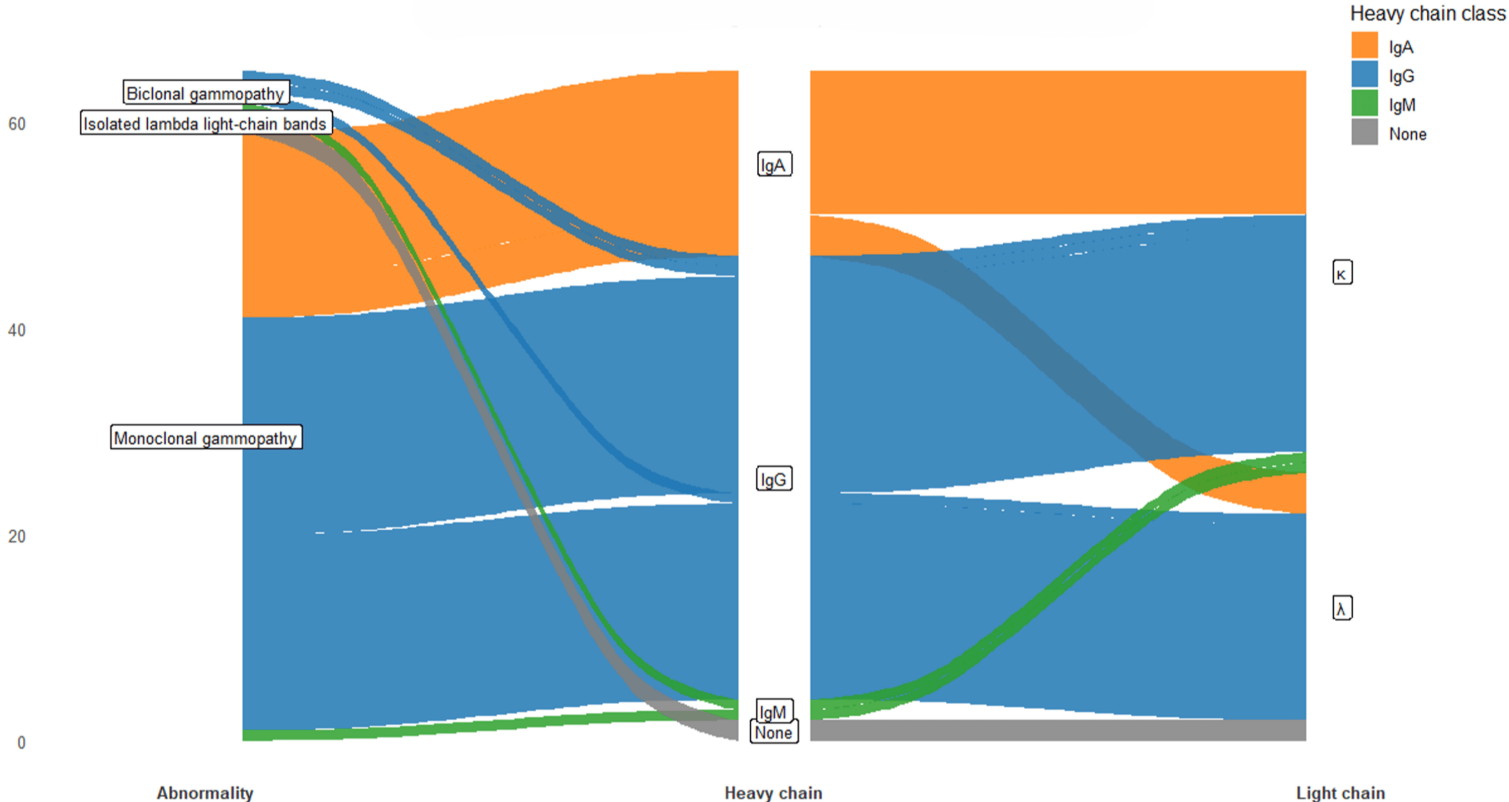
Flow of immunofixation abnormalities from abnormality type to heavy and light chains. Sankey-style representation of monoclonal, biclonal, and isolated light-chain abnormalities by heavy-chain (IgG, IgA, and IgM) and light-chain (κ, λ) class. Figure created by the authors with RStudio (Posit PBC, USA)

Two patients (3.1%) displayed biclonal patterns, one combining IgG-κ and IgM-κ and the other IgG-κ and IgG-λ, indicating dual clonal plasma-cell populations (Figure 3). In addition, two patients (3.1%) exhibited isolated λ light-chain bands without a corresponding heavy-chain component, prompting further urinary immunofixation testing for Bence-Jones proteinuria, consistent with international diagnostic recommendations.

Overall, serum immunofixation revealed a clinically relevant monoclonal or oligoclonal component in approximately one quarter of hypogammaglobulinemic patients, demonstrating its high diagnostic yield even in the context of low total γ-globulin concentrations. The predominance of IgG-type monoclonalities aligns with literature reporting IgG as the most frequent class in monoclonal gammopathies, while the lesser frequency of IgA and IgM clones reflects their known epidemiological distribution.

The detection of biclonal and light-chain-only patterns further highlights the sensitivity of IFE in uncovering complex or evolving plasma-cell disorders. Conversely, the large proportion of negative IFE results (75.9%) suggests that most hypogammaglobulinemias in this hospital cohort were likely secondary or polyclonal, associated with conditions such as chronic infection, autoimmune disease, or iatrogenic immunosuppression.

## Discussion

The present study provides a comprehensive evaluation of serum IFE in patients presenting with hypogammaglobulinemia, a biochemical abnormality that may conceal early or atypical plasma-cell dyscrasias. Our findings highlight that nearly one quarter (24.1%) of patients with decreased γ-globulin levels (<8 g/L) exhibited a monoclonal or oligoclonal component detectable exclusively by IFE, emphasizing the test’s superior analytical sensitivity compared with conventional SPEP. This observation is consistent with previous investigations showing that 15-25% of patients with normal or flattened electrophoretic γ-globulin zones harbor low-concentration monoclonal proteins identifiable only through IFE or FLC analysis [16,17]. The predominance of IgG monoclonal isotypes (67.8%), followed by IgA (30.5%) and IgM (1.7%), aligns closely with the distribution patterns established in large epidemiological cohorts of monoclonal gammopathies. In population-based studies from the Mayo Clinic and the SEER registries, IgG clones account for approximately 60-70% of MGUS and multiple myeloma cases, IgA for 15-25%, and IgM for 5-10% [18,19]. This hierarchy reflects the underlying biology of plasma-cell differentiation and class switching, as well as the higher stability and longer serum half-life of IgG molecules. The close concordance between our findings and these international benchmarks reinforces the clinical representativeness and external validity of our cohort.

The identification of biclonal gammopathies in 3.1% of cases is also in line with reported frequencies of 1-3% in major series [20,21]. Biclonal patterns most often involve combinations of IgG and IgA or two IgG subclasses and typically result from either two independent plasma-cell clones or a single clone undergoing class-switch recombination. Although biclonality does not necessarily predict an adverse prognosis, its recognition has important diagnostic implications, as one clone may evolve independently toward malignant transformation [22].

Of particular clinical relevance were two cases (3.1%) of isolated λ light-chain bands, which could represent light-chain MGUS, early light-chain myeloma, or AL amyloidosis. Such profiles are frequently missed on standard electrophoresis but may be detected by IFE due to its ability to identify free κ or λ chains in the absence of a heavy-chain component. Current guidelines from the International Myeloma Working Group (IMWG) emphasize that in suspected plasma-cell disorders, the combination of serum IFE, urine IFE, and FLC assay provides the highest diagnostic sensitivity for detecting both intact and light-chain-only monoclonal components [23,24]. Consequently, reflex testing for Bence-Jones proteinuria remains warranted when isolated light-chain bands are encountered, as performed in our study.

Conversely, the majority of patients in our cohort (75.9%) exhibited negative serum immunofixation results, consistent with the interpretation that most hypogammaglobulinemias encountered in tertiary-care settings are secondary or polyclonal rather than clonal in origin. Secondary hypogammaglobulinemia results from heterogeneous mechanisms leading to either impaired Ig synthesis or excessive Ig loss. Common etiologies include chronic infections (e.g., HIV, hepatitis, or tuberculosis), autoimmune and inflammatory disorders such as systemic lupus erythematosus or rheumatoid arthritis, and protein-losing conditions such as nephrotic syndrome and protein-losing enteropathy [25-27].

In hospital populations, iatrogenic causes are increasingly recognized, particularly due to the widespread use of immunosuppressive therapies, corticosteroids, cytotoxic chemotherapy, and B-cell-depleting monoclonal antibodies such as rituximab. These agents cause transient or persistent hypogammaglobulinemia by suppressing plasma-cell differentiation and reducing Ig production [28]. Similarly, targeted therapies for autoimmune and malignant diseases, such as anti-CD38 (daratumumab) and BTK inhibitors (ibrutinib), have been implicated in acquired antibody deficiency syndromes [26].

Therefore, while a negative IFE substantially reduces the likelihood of a monoclonal gammopathy, low-level or non-secretory disease may still be missed, and clinical concern may persist. Persistent or severe hypogammaglobulinemia in such patients warrants evaluation for underlying immunoregulatory defects or secondary causes, particularly when accompanied by recurrent or opportunistic infections. This reinforces the dual diagnostic role of the clinical biochemistry laboratory, not only in detecting clonal plasma-cell processes through IFE, but also in identifying polyclonal immune suppression or iatrogenic antibody deficiencies that carry significant clinical implications [25,27].

Collectively, our findings reaffirm that hypogammaglobulinemia should not be viewed solely as a quantitative immunodeficiency, but also as a possible presentation of subtle plasma-cell or lymphoplasmacytic disorders. Serum IFE thus remains indispensable for (1) confirming secretory clonal activity, (2) characterizing heavy- and light-chain isotypes, (3) identifying biclonal patterns, and (4) detecting isolated light-chain abnormalities invisible on SPEP. From a clinical-laboratory standpoint, incorporating IFE, and, when indicated, FLC and urine IFE, into the standard hypogammaglobulinemia work-up substantially enhances early recognition of clonal processes, facilitates hematologic referral, and supports precision monitoring of disease evolution.

Study limitations

Several limitations should be acknowledged. This retrospective, descriptive, single-center study conducted in a tertiary referral hospital limits causal inference and generalizability beyond our setting. Because most requests originated from hematology/internal medicine/oncology, the cohort was likely referral-enriched, introducing potential selection bias. Analyses were descriptive only, without a comparator group or adjustment for clinical confounders, and follow-up was not consistently available to assess the longer-term significance of detected monoclonal bands. Finally, defining hypogammaglobulinemia as γ-globulin <8 g/L may have included milder cases and influenced the observed detection rate.

## Conclusions

Hypogammaglobulinemia should not be considered only a marker of immune deficiency or protein loss; it can also conceal an underlying monoclonal process that may be missed on SPEP. In our cohort, serum immunofixation identified clonal abnormalities in 24.1% of patients, showing that low γ-globulin levels do not exclude, and may even mask, a secretory clone.

Serum immunofixation proved essential because of its higher sensitivity for low-level monoclonal proteins, its ability to define heavy- and light-chain isotypes, detect biclonal patterns, and reveal light-chain-only abnormalities (including isolated λ bands, prompting urine immunofixation when appropriate). These findings support routinely integrating serum immunofixation into the initial evaluation of hypogammaglobulinemia, rather than reserving it for hypergammaglobulinemia or overtly suspicious SPEP patterns.
